# Knowledge, awareness, and risk practices related to bacterial contamination of antiseptics, disinfectants, and hand hygiene products among healthcare workers in sub-saharan Africa: a cross-sectional survey in three tertiary care hospitals (Benin, Burkina Faso, and DR Congo)

**DOI:** 10.1186/s13756-024-01396-3

**Published:** 2024-04-16

**Authors:** Palpouguini Lompo, Anne-Sophie Heroes, Kadija Ouédraogo, Patient Okitale, Abel Wakpo, Jocelyne Kalema, Octavie Lunguya, Halidou Tinto, Dissou Affolabi, Lassana Sangaré, Jan Jacobs

**Affiliations:** 1 Clinical Research Unit of Nanoro, Institut de Recherche en Science de la Santé, Ouagadougou, 11 BP 218 Burkina Faso; 2 Department of Clinical Sciences, Institute of Tropical Medicine, Nationalestraat 155, Antwerp, 2000 Belgium; 3 Department of Microbiology, Immunology and Transplantation, KU Leuven, Naamsestraat 22, Box 5401, Leuven, 3000 Belgium; 4 Département de Microbiologie, Cliniques Universitaires de Kinshasa, BP 127, Kinshasa, Congo; 5 Centre National Hospitalier Universitaire Hubert Koutoukou Maga, Cotonou, 01 BP 386 Benin; 6 Département de Microbiologie, National Institute of Biomedical Research, Av. De la Démocratie N°5345, Kinshasa, Congo; 7 Centre Hospitalier Universitaire Yalgado Ouédraogo, Ouagadougou, 03 BP 7022 Burkina Faso

**Keywords:** Bacteria, Contamination, Antiseptics, Disinfectants, Hand hygiene, Knowledge, Awareness, Practice

## Abstract

**Background:**

Antiseptics, disinfectants, and hand hygiene products can be contaminated with bacteria and cause healthcare-associated infections, which are underreported from low- and middle-income countries. To better understand the user-related risk factors, we conducted a knowledge, awareness, and practice survey among hospital staff in sub-Saharan Africa.

**Methods:**

Self-administered questionnaire distributed among healthcare workers in three tertiary care hospitals (Burkina Faso, Benin, Democratic Republic of the Congo).

**Results:**

617 healthcare workers (85.3% (para)medical and 14.7% auxiliary staff) participated. Less than half (45.5%) had been trained in Infection Prevention & Control (IPC), and only 15.7% were trained < 1 year ago. Near two-thirds (64.2%) preferred liquid soap for hand hygiene, versus 33.1% for alcohol-based hand rub (ABHR). Most (58.3%) expressed confidence in the locally available products. Knowledge of product categories, storage conditions and shelf-life was inadequate: eosin was considered as an antiseptic (47.5% of (para)medical staff), the shelf life and storage conditions (non-transparent container) of freshly prepared chlorine 0.5% were known by only 42.6% and 34.8% of participants, respectively. Approximately one-third of participants approved using tap water for preparation of chlorine 0.5% and liquid soap. Most participants (> 80%) disapproved recycling soft-drink bottles as liquid soap containers. Nearly two-thirds (65.0%) declared that bacteria may be resistant to and survive in ABHR, versus 51.0% and 37.4% for povidone iodine and chlorine 0.5%, respectively. Depicted risk practices (*n* = 4) were ignored by 30 to 40% of participants: they included touching the rim or content of stock containers with compresses or small containers, storing of cotton balls soaked in an antiseptic, and hand-touching the spout of pump dispenser. Filling containers by topping-up was considered good practice by 18.3% of participants. Half (52.1%) of participants acknowledged indefinite reuse of containers. Besides small differences, the findings were similar across the study sites and professional groups. Among IPC-trained staff, proportions recognizing all 4 risk practices were higher compared to non-trained staff (35.9% versus 23.8%, *p* < 0.0001).

**Conclusions:**

The present findings can guide tailored training and IPC implementation at the healthcare facility and national levels, and sensitize stakeholders’ and funders’ interest.

**Supplementary Information:**

The online version contains supplementary material available at 10.1186/s13756-024-01396-3.

## Introduction

Healthcare-associated infections cause a considerable burden of mortality, morbidity, and economic loss [1] and the healthcare environment constitutes a reservoir of multidrug resistant bacteria [2–4]. Despite the paucity of data, low- and middle-income countries (LMIC) including sub-Saharan Africa, face the highest incidence and burden of healthcare-associated infections [1, 5, 6].

Among the objects in the hospital environment are antiseptics, disinfectants and products used for hand hygiene (AS, DI and HH products). Antiseptics (e.g., ethanol, chlorhexidine) and disinfectants (e.g., chlorine) inactivate microorganisms or inhibit their growth; they are applied on skin or mucous membranes (antiseptics) [7] and on objects and surfaces (disinfectants) [8]. Products used for hand hygiene are alcohol-based hand rub (ABHR) and liquid and bar soap; soap can contain antiseptics (antiseptic soap) or not (plain soap) [9].

Antiseptics and disinfectants are vulnerable to bacterial contamination too, mostly by Gram-negative bacteria [10, 11]. This possibility is often overlooked but contamination of AS, DI and HH products has been documented in cross-sectional studies and been implicated in numerous healthcare-associated outbreaks, but with considerable underreporting from LMIC [3, 12, 13]. Despite an anticipated decline [13], published reports have persisted during past decades and in addition reports of contaminated liquid soap have emerged [11, 14].

Factors conducive to bacterial contamination of AS, DI and HH products are numerous and intertwined: they are frequently co-present in LMIC [11, 14]. Water-based products are more vulnerable to contamination, particularly if (over)diluted or diluted with non-sterile water, like tap water [11, 14]. Further, some contaminating bacteria (mainly Gram-negative bacteria) are intrinsically (naturally) resistant to certain products. Moreover, they may produce so-called biofilms, which are defined as an association of bacterial cells, which may belong to different species, with an extracellular polymeric substance matrix shielding them from external threats such as drying, starvation or antimicrobials [15, 16].

In LMIC, given lack of access or affordability, reuse of containers is common. The definition of the terms “reused” and “recycled” in this article is purposely adopted from a previously published study [10]. Many reused containers were originally disposable containers of a branded product. These forms or reused containers are further named “recycled containers”. Even soft-drink bottles may be recycled as liquid soap containers. The reuse of containers requires reprocessing: this includes careful emptying, cleaning with safe water *(e.g.*., distilled, or freshly preboiled water) and sterilization or disinfection [17–19]. Sterilization by autoclaving is the preferred method, alternately, the method used for baby milk bottles consisting to soak the containers in a boiling water, 100 °C. If not possible, the containers should be soaked in a biofilm-active agent [19] such as chlorine 0.5%, after which abundant rinsing with safe water and subsequent drying is needed. Most recycled product containers (disposable container which was originally used for another product) and soft-drink bottles are made from high density polyethylene (HDPE) and respectively polyethylene terephthalate (PET), which do not withstand autoclaving [14]. Furthermore, reprocessing is often only partially or not at all done [14].

Further, inappropriate end-user practices may contribute to contamination: examples are soaking cotton balls in antiseptics, and refilling containers without first emptying and reprocessing them (the latter is termed “topping-up” [11]). Behind these inappropriate practices are the incorrect assumptions that AS, DI and HH products eradicate all bacteria [20–22] and are sterile [23, 24].

In a recent microbiological study of AS, DI and HH products in two university hospitals in West-Africa (CHU-YO and CNHU-HKM), we observed inappropriate practices and apparent low-risk awareness about contamination [10]. To better understand these observations and their extent, we organized a survey among the healthcare staff of these hospitals and in a third hospital in Central Africa. The objectives were to assess the knowledge, awareness, and practices related to the risk of bacterial contamination of AS, DI and HH products.

## Materials and methods

### Study design: study sites, participants, and period

The survey consisted of a self-administered questionnaire and was conducted in three tertiary care hospitals: Centre Hospitalier Universitaire Yalgado Ouédraogo in Ouagadougou, Burkina Faso (CHU-YO), Centre National Hospitalier Universitaire Hubert Koutoukou Maga, Cotonou, Benin (CNHU-HKM), University Hospital of Kinshasa (UHK), Kinshasa, Democratic Republic of the Congo (DR Congo). All three hospitals are university teaching hospitals. Burkina Faso and Benin are respectively a low- and a middle-income country in West Africa, DR Congo is a low-income country in Central Africa. In CHU-YO and CNHU-HKM, a microbiological survey of AS, DI and HH products was conducted prior to the study. As part of this microbiological survey, interviews and observations were done to understand the procurement, preparation, distribution, and end-user practices related to the products [10]. These brief interviews were also conducted later in UHK. The findings from the interviews were used to develop the survey questionnaire.

Eligible participants were healthcare workers using AS, DI and HH products. The sample size was calculated with an online calculator [25]. For a target population of 400 healthcare workers (i.e., the total number of healthcare workers at CHU-YO in Burkina Faso), a confidence level of 95%, a margin of error of 5% and a response distribution of 50% were applied, resulting in a sample size of 197 completed questionnaires. For the other sites, a similar sample size was used.

Questionnaires were administered in October and November 2020 in Burkina Faso, from January to May 2021 in DR Congo, and from December 2021 to March 2022 in Benin.

### Questionnaire: structure, selection and format of questions, piloting

The questionnaire was drafted by the principal investigator (PL) and co-author A-SH and commented for content, context-adapted formulation and clarity and readability by co-authors KO and JJ. It consisted of multiple-choice questions inspired by relevant references from a literature review [14], and by findings of the microbiological survey of AS, DI and HH products in CHU-YO and CNHU-HKM [10].

An English-language draft version of the questionnaire was piloted in 2019 among medical doctors, nurses, pharmacists, and laboratory technicians from LMICs participating in a short course on the containment of antimicrobial resistance in hospitals in low-resource settings [26]. Next, it was translated to French by PL and KO.

Product names used in the survey were those used in daily practice by the participants and included proper names and jargon names (Table 1). An example is the liquid chlorine-based disinfectant referred to as “Eau de Javel”, which is the French name equivalent of household bleach. As the name “Eau de Javel” in all three hospitals also referred to the 0.5% prepared working solution, the name was used to denote the working solution; in the article text, the term chlorine 0.5% is used. Although no longer recommended for routine use in healthcare [7], bar soap was included in the questionnaire as it was observed to be in use at CHU-YO during the microbiological survey [10].

**Table 1:** Product names used in the survey questionnaire assessing healthcare workers’ knowledge, awareness, and practices

Surveyed products	Country Model List of Essential Medicine [27–29]
Antiseptics	
• Betadine (**povidone-iodine 10%**), in the text referred to as povidone iodine	Burkina Faso, Benin, DR Cogo
• **ethanol 70%**	Burkina Faso: ethanol 70% Benin: alcohol 70% (type of alcohol not mentioned) DR Congo: listed as antiseptic and as disinfectant
• Dakin solution (stabilized chlorine product)	Burkina Faso: “Stabilized sodium hypochlorite”
• isopropanol 70% (isopropyl alcohol 70%), in the text referred to as isopropyl alcohol 70%	Not listed
Disinfectants	
• *Eau de Javel* 0.5% (**chlorine-based compound**) *, in the text referred to as chlorine 0.5%	Burkina Faso: sodium or calcium hypochlorite Benin: sodium hypochlorite
Hand hygiene products	
• **alcohol-based hand rub** (ABHR, listed as disinfectant in the WHO EML [30, 31]	Not listed
• antiseptic liquid soap: contains an antiseptic	Not listed
• plain liquid soap: liquid soap without antiseptic	Not listed
• bar soap	Not listed

*WHO EML mentions for liquid chlorine a concentration of 0.1%. Other WHO documents mention concentrations of 0.5% for environmental disinfection [32]

Product names were those used in daily practice by the participants and included proper names (capitalized) and jargon names (italicized), for which the active ingredient is written between brackets. Products mentioned in bold are listed in the World Health Organization Model List of Essential Medicines (abbreviated as WHO EML) [30, 31]. When different from those listed in the table, the product names used in the body text are added. The column at the right indicates if the products are listed on the countries’ Medicines. Abbreviation: DR Congo = Democratic Republic of the Congo

As the survey was hospital-wide, it only addressed hand hygiene and not surgical hand preparation. Based on participants’ feedback and analysis of the first results, the initial Burkina Faso version was slightly adapted to the settings of DR Congo and Benin. Changes were mainly related to comprehensibility (e.g., adding between brackets the word “microbes” next to “bacteria”).

As two questions were removed from analysis (see below), the final questionnaire consisted of 31 questions. Next to sociodemographic and professional information, usage and user preferences for products and containers were surveyed. Table 2 presents the knowledge, awareness and practice categories and the different items surveyed. The detailed questions and the supporting references can be found in Supplementary Table 1. The numbers of answer options per question ranged from three to eight per question. For 19 questions, selecting multiple options was possible.

### Recruitment of participants and administration of the questionnaire

At CHU-YO (Burkina Faso), the study staff conducting the survey consisted of the principal investigator (PL) and a social scientist (KO). At CNHU-HKM (Benin) and UHK (DR Congo), the study staff consisted of trained local medical doctors. During the morning of the survey days, the study teams visited the wards and informed the healthcare workers present in the wards about the survey. They were invited to present later that day to a focal meeting point where they received instructions in small groups (due to COVID-19 restrictions), and filled in the questionnaire on site.

The questionnaire was formatted in Kobo Toolbox (Kobo Collect v1.25.1, v1.28.0–10 and v2022.1.2 (Kobo, Massachusetts, US)) [33] and presented on tablet computers. During on-site filling-in of the questionnaire, study staff were available for guidance. Based on the pilot testing, the process time for filling-in the survey had been estimated at 30 min. Data were collected over 22 to maximum 49 days per site. Filling-in was done offline; the completed questionnaires were uploaded daily to the Kobo Toolbox server.

Questions appeared one by one on the tablet computer. Once a participant confirmed his/her response, he/she was not able to return and correct previous responses. Skipping questions and returning to previous questions were not possible, and the questionnaire could not be stopped before completion.

**Table 2:** Items covered by the questionnaire used to assess healthcare workers’ knowledge, awareness, and practices

**Fig. 1:**
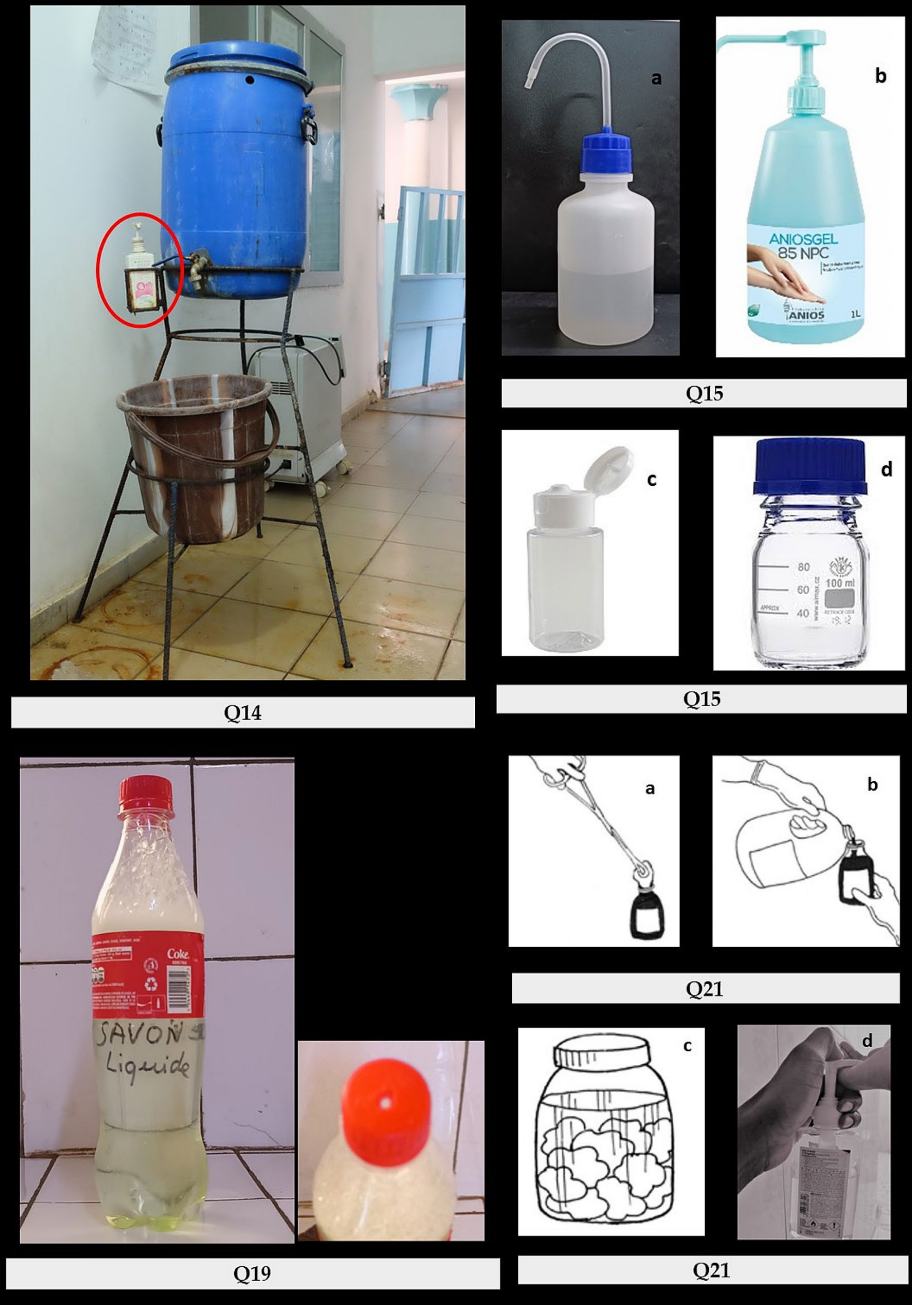
Pictures illustrating the photo-based questions of the survey

Items covered by the questionnaire
**Demographics, professional profile of participants**: age, gender, profession, hospital/center, ward (see Table 3)
**Familiarity with AS, DI and HH products** (see Table 3)
Experience with AS, DI and HH products
Training in IPC (≤ 1 year ago, > 1 year ago, never, or not recalled)
Confidence in products used in the ward
Preference of products used for hand hygiene
Products actually used for hand hygiene in the ward
Preference for container types (alcohol-based hand rub) (see Fig. 1)
**Knowledge about bacterial contamination**
Knowledge about products used for skin disinfection
Knowledge about products used for disinfection of equipment and surfaces
Vulnerability to bacterial contamination: povidone iodine, alcohol-based hand rub, chlorine 0.5%, household soap
Period-after-opening (example of povidone iodine)
Shelf life of chlorine 0.5% solution and ethanol 70%
Storage of chlorine 0.5% solution versus ethanol 70% (transparent container)
**Awareness of the risk of bacterial contamination**
Preparation of alcohol and chlorine products in the pharmacy: use of tap water
Preparation of chlorine products in the pharmacy: cleaning of utensils
Use of liquid soap, mobile handwash station (see Fig. 1): tap water, period-after-opening, reprocessing of containers
Recognizing depicted risk practices when handling products and containers (see Fig. 1)
Acceptability of recycling a soft-drink bottle as liquid soap container (see Fig. 1)
Recognizing bar soap risk contamination mitigation practices: use of small pieces, use receptacle allowing drainage of fluid
Practice of “topping-up” soap containers in a high-risk ward (neonatal ward)
**Risk Practices conducive to bacterial contamination**
Reuse of containers: frequency
Reprocessing of containers: how are containers reprocessed?

Abbreviations: AS = antiseptics, DI = disinfectants, HH = hand hygiene

### Data analysis

Data from the Kobo Toolbox server were exported to an Excel file (Microsoft, Redmond, WA USA) for analysis. Professional profiles of participants were grouped as (i) (para)medical staff (subgroups medical doctors and trainees, nursing and midwife staff, pharmacists, and laboratory staff) and (ii) auxiliary staff. Auxiliary staff were non-medically qualified support staff assisting in patient or sample transport, housekeeping, cleaning-up spills, and cleaning of patients’ rooms. Participants who replied they had never used AS, DI or HH products were removed from analysis. In retrospect, two questions were removed from analysis: one question about recycling of containers (because it missed one answer option), and another about the recommended concentration of ethanol in ABHR (which was 70% at all three sites, i.e., different from the 80% recommended by WHO [7, 30, 31].

For most questions categorized as knowledge, awareness, and practice, expected correct options were defined according to published evidence (Supplementary Table 1). Unless otherwise stated, frequencies of answered options were displayed on the total number of participants. Results were presented for all participants aggregated; in case of substantial differences, results per site or per (sub)group were presented. Differences in proportions were assessed by the Pearson chi-square test or Fisher’s Exact test, and considered significant at a *p*-value < 0.05.

### Post-hoc internal validity check of the questionnaire

As a post-hoc validity check, the frequency of invalid answers was assessed. Invalid answers consisted of nonsense options such as “I do not know” combined with another option. The acceptance threshold was set at < 5%. Congruence was assessed by comparing answers to questions addressing similar items.

## Results

### Study sites

Pre-survey interviews confirmed that UHK had no implemented procedure about hand hygiene. Neither procedures about the use of AS, DI and HH products nor the reprocessing of containers were available, as was previously noted for the two other hospitals [10].

### Demographics and professional profile of the participants

Overall, among the 633 healthcare workers presenting at the survey’s meeting points, 617 (97.5%) participated in the survey; 45.5% were female, and 62.6% were ≤ 40 years old (Table 3). The (para)medical staff comprised 85.3% of participants with medical doctors and nursing & midwife staff each representing approximately 40% of participants. Auxiliary staff accounted for 14.7% of participants. The most represented wards were surgery, maternity, and pediatrics including neonatology. The median (range) time to complete the survey was 21 (2–134) minutes. Long durations (> 60 min, observed in 26 participants) were all due to duty-related interruptions.

### Acquaintance with AS, DI and HH products: experience and training

As to product experience, most participants indicated hand hygiene (Table 3). Wound care, surface cleaning and device cleaning (stethoscope, thermometer) were indicated by two-thirds of participants for each. Most auxiliary staff, but also two-thirds of (para)medical staff, declared experience with surface cleaning. Preparation and procurement were indicated by approximately 15% of participants for each. Overall, less than half (45.5%) of participants had been trained in Infection Prevention and Control (IPC), one-third of them (15.7% of all participants) had been trained during the year before the survey. Medical doctors (including trainees and students) had the lowest proportion of training; nearly two-thirds of them at CHU-YO (63.9%) and CNHU-HKM (65.7%) had never attended an IPC training.

### Participants’ experiences with the products: preferences and confidence

When asked for their preferred product for hand hygiene (question with one option for answer), one-third (33.1%) of participants indicated ABHR, whereas nearly two-thirds (64.3%) indicated liquid soap (either antiseptic or plain soap); only 1.8% preferred bar soap (Table 3). (Para)medical staff more frequently preferred ABHR compared to auxiliary staff (35.9% versus 25.3%, respectively, *p* = 0.05). These patterns were consistent across all three study sites. Preferences for ABHR were higher (45.5%) at CHU-YO compared to < 30% at CNHU-HKM and UHK. When questioned about the products they used for hand hygiene (multiple options could be selected), ABHR and liquid soap were chosen by 67.9% and 85.3% of participants. Nearly 60% of participants declared they had confidence in the quality of locally available products; close to one third (32.3%) mentioned some confidence. Across all three study sites, auxiliary staff more frequently expressed confidence than (para)medical staff (79.1% versus 54.8%, *p* < 0.0001).

**Table 3:** Overview of the participants’ demographics and answers to selected questions

Items	CHU-YO (*n* = 200)		CNHU-HKM (*n* = 229)	CUK (*n* = 188)		Total (*n* = 617)	
**Staff presenting for the questionnaire**	**201**		**235**	**197**		**633**	
No consent given	0		4	8		12	
Never used AS DI and HH products	1		2	1		4	
Staff who completed the survey	200 (99.5%)		229 (97.4%)	188 (95.4%)		617 (97.5%)	
**Female gender**	98 (49.0%)		100 (43.7%)	83 (44.1%)		281 (45.5%)	
**Age**							
< 18 years	0		0	1 (0.5%)		1 (0.2%)	
18–30 years	66 (33.0%)		91 (39.7%)	40 (21.3%)		197 (31.9%)	
31–40 years	42 (21.0%)		72 (31.4%)	74 (39.4%)		188 (30.5%)	
41–50 years	65 (32.5%)		56 (24.5%)	42 (22.3%)		163 (26.4%)	
> 50 years	27 (13.5%)		10 (4.4%)	31 (16.5%)		68 (11.0%)	
**Professional profile**							
(Para-)medical staff	179 (89.5%)		183 (79.9%)	164 (87.2%)		526 (85.3%)	
Medical doctors and students	61 (30.5%)		108 (47.2%)	86 (45.7%)		255 (41.3%)	
Nurses, midwife and students	116 (58.0%)		71 (31.0%)	58 (30.9%)		245 (39.7%)	
Pharmacist and lab staff	2 (1.0%)		4 (1.7%)	20 (10.6%)		26 (4.2%)	
Auxiliary staff	21 (10.5%)		46 (20.1%)	24 (12.8%)		91 (14.7%)	
**Hospital ward**							
Maternity	63 (31.5%)		42 (18.3%)	35 (18.6%)		140 (22.7%)	
Pediatrics	41 (20.5%)		27 (11.8%)	18 (9.6%)		86 (13.9%)	
Neonatology	13 (6.5%)		25 (10.9%)	11 (5.9%)		49 (7.9%)	
Internal medicine	30 (15.0%)		62 (27.1%)	36 (19.1%)		128 (20.7%)	
Surgery	47 (23.5%)		71 (31.0%)	26 (13.8%)		144 (23.3%)	
Pharmacy and laboratory	2 (1.0%)		1 (0.4%)	30 (16.0%)		33 (5.3%)	
Other	4 (2.0%)		1 (0.4%)	32 (17.0%)		37 (6.0%)	
**Training in Infection Prevention and Control**							
≤ 1 year,	28 (14.0%)		39 (17.0%)	30 (16.0%)		97 (15.7%)	
> 1 year ago,	66 (33.0%)		56 (24.5%)	62 (33.0%)		184 (29.8%)	
Never attended/could not recall	106 (53.0%)		134 (58.5%)	96 (51.1%)		336 (54.5%)	
**Experience with antiseptics and disinfectants** (multiple options could be selected)							
Hand hygiene	190 (95.0%)		219 (95.6%)	173 (92.0%)		582 (94.3%)	
Skin antisepsis	135 (67.5%)		117 (51.1%)	100 (53.2%)		352 (57.1%)	
Wound care	149 (74.5%)		161 (70.3%)	93 (49.5%)		403 (65.3%)	
Medical device disinfection	143 (71.5%)		150 (65.5%)	116 (61.7%)		409 (66.3%)	
Surface cleaning	140 (70.0%)		148 (64.6%)	122 (64.9%)		410 (66.5%)	
AS and DI preparation	23 (11.5%)		34 (14.8%)	42 (22.3%)		99 (16.0%)	
AS and DI procurement	20 (10.0%)		28 (12.2%)	29 (15.4%)		77 (12.5%)	
**Preference for hand hygiene product container** (multiple options could be selected)							
Table-top pump dispenser container	180 (90.0%)		189 (82.5%)	173 (92.0%)		541 (87.7%)	
Squeeze bottle	45 (22.5%)		66 (28.8%)	33 (17.6%)		144 (23.3%)	
Snap-cap dropper container	22 (11.0%)		54 (23.6%)	54 (28.7%)		130 (21.1%)	
Screw cap container	3 (1.5%)		4 (1.7%)	15 (8.0%)		22 (3.6%)	
**Confidence in products used in the ward**							
Confident– (all staff) *	117 (58.5%)		130 (56.8%)	113 (60.1%)		360 (58.3%)	
(Para)medical staff	103 (57.5%)		94 (51.4%)	91 (55.5%)		288 (54.8%)	
Auxiliary staff	14 (66.7%)		36 (78.3%)	22 (91.7%)		72 (79.1%)	
A bit confident (all staff)	71 (35.5%)		72 (31.4%)	56 (29.8%)		199 (32.3%)	
Not confident (all staff)	12 (6.0%)		27 (11.8%)	19 (10.1%)		58 (9.4%)	
**Preference of product used for hand hygiene** (only one option could be selected)							
ABHR	91 (45.5%)		60 (26.2%)	53 (28.2%)		204 (33.1%)	
Liquid soap **	103 (51.5%)		164 (71.6%)	129 (68.6%)		396 (64.2%)	
Bar soap	5 (2.5%)		2 (0.9%°	4 (2.1%)		11 (1.8%)	
No preference	1 (0.5%)		2 (0.9%)	0		3 (0.5%)	
**Products actually used for hand hygiene in the ward** (multiple options could be selected)							
ABHR	136 (68.0%)		162 (70.7%)	121 (64.4%)		418 (67.7%)	
Liquid soap **	136 (68.0%)		213 (93.0%)	177 (94.1%)		526 (85.3%)	
Any of the products	26 (13.0%)		22 (9.6%)	19 (10.1%)		67 (10.9%)	
I do not know	3 (1.5%)		1 (0.4%)	0		4 (0.6%)	

*including “*I am absolutely confident*” and “*I am confident*”

** Liquid soap combines antiseptic and plain liquid soap

Numbers are presented for all participants combined unless otherwise stated. Abbreviations: ABHR = Alcohol-based Hand Rub, AS = antiseptics, DI = disinfectants, HH = hand hygiene. CHU-YO = Centre Hospitalier Universitaire Yalgado Ouédraogo (Burkina Faso), CNHU-HKM = Centre National Hospitalier Universitaire Hubert Koutoukou Maga (Benin), UHK = University Hospital of Kinshasa, Kinshasa (DR Congo)

Out of four different types of containers (Fig. 1, multiple options for answer possible), most (87.8%) of participants expressed a preference for the table-top container with hand-commanded pump dispenser. Squeeze bottle and container with a snap-cap dropper ranked close to each other as second and third choice, respectively, and with much lower preferences. The screw-cap container yielded a very low preference (Table 3).

### Knowledge about products and their category (antiseptic versus disinfectant)

Products correctly identified as antiseptics were povidone iodine (85.1% of participants) and Dakin solution (74.9%, although lower in UHK (59.6%)). Ethanol 70% and isopropyl alcohol 70% were correctly identified as antiseptics by less than half (43.9%) and a few (3.1%) participants, respectively. Methanol and glycerol were (incorrectly) assigned as antiseptics by only a few participants (2.8% and 9.8%, respectively) whereas chlorine 0.5% and eosin were incorrectly considered as antiseptics by respectively 29.8% and 43.3% of participants, the latter most frequently (47.5%) among (para)medical staff.

Question (Q) 14, mobile hand-washing station, questioning the manufacturing of liquid soap (red circle) with tap water, its in-use stability, and the reprocessing of the container. Q15 assessed the preferred type of container for alcohol-based handrub: squeeze bottle (a), table-top container with pump dispenser (b), container with flip-top snap-cap dropper (c) and screw-cap container (d). Q19, assessed the acceptability of recycling a soft drink bottle as container for liquid soap. Q21 showed practices at risk for contamination when handling containers: impregnating a cotton pad by touching the container’s rim (a), filling a small container with a large stock container while touching the rims of both containers (b), storage of cotton pads soaked in a container with antiseptic (c) and hand-touching the spout of the dispenser’s pump while dispensing products for hand hygiene (d). Figure 21a, b and c were reproduced from reference [34] with permission.

Most participants (93.5%) recognized chlorine 0.5% as a disinfectant. Less frequently considered as a disinfectant were ethanol 70% and isopropyl alcohol 70% (34.5% and 11.2%, respectively). Dakin solution and povidone iodine were incorrectly considered as disinfectants by 22.7% and 11.8% of participants, with the largest proportions respectively at CNHU-HKM (46.3%) and UHK (17.6%). Glycerol, eosin, and methanol were incorrectly replied as disinfectants by less than 10% of participants each.

### Knowledge about products’ vulnerability to bacterial contamination

Nearly half and a quarter of participants considered that chlorine 0.5% and povidone iodine “*kills all bacteria*”. For ABHR and household soap, proportions of participants were less than 20% and 10%, respectively (Table 4). Conversely, in decreasing proportion, participants ranked susceptibility to contamination (“*bacteria are resistant to… and can survive*”) as follows: household soap (considered as vulnerable by over 70% of participants), ABHR (two-thirds of participants), povidone iodine (half of participants), and chlorine 0.5% (37.4% of participants). For the question “*bacteria can multiply in the product*”, a similar rank and lower proportions were noted. The ranking of vulnerability was consistent across the study sites and similar among professional groups, apart from an inverse ranking of ABHR and povidone iodine among the auxiliary staff.

### Knowledge about storage, shelf life/expiry date, and period-after-opening

When questioned about the shelf life of freshly prepared chlorine 0.5%, 42.6% of participants correctly replied to use the product within a maximum of 1 day, the other participants declared not to know the answer (25.6%), or replied longer periods (up to > 1 week). Auxiliary staff replied considerably better compared to (para)medical staff (67.0% versus 38.4%, *p* < 0.0001). For the in-use stability of locally manufactured liquid soap at a mobile handwash station (Fig. 1), only one third of participants (30.3%) agreed with the proposed period of maximum 1 week. The lowest and largest proportions were noted among nursing staff at CNHU-HKM (9.9%) and auxiliary staff at UHK (66.7%), respectively. A photo-/case-based question assessed the period-after-opening (in-use stability time). It depicted an in-use branded container with povidone iodine, within expiry date but already opened and in-use for seven months. Half (53.5%) of participants declared not to use it, 40.7% acknowledged they would still use the product, and the remaining declared not to know it.

**Table 4:** Participants’ answers to the questions about the vulnerability of products to bacterial contamination

Products	Sites (nr. of participants)				Groups (nr. of participants)		Total (*n* = 617)
	CHU-YO (*n* = 200)	CNHU-HKM (*n* = 229)	UHK (*n* = 188)		(Para)medical (*n* = 526)	Auxiliary (*n* = 91)	
**Some bacteria are resistant and can survive in the product**							
Chlorine 0.5%	38.4%	40.3%	33.0%		39.8%	22.9%	37.4%
Povidone iodine 10%	62.2%	51.8%	38.0%		54.8%	30.2%	51.0%
ABHR	70.8%	67.3%	56.0%		70.4%	30.5%	65.0%
Household soap	75.1%	70.7%	65.6%		71.5%	65.2%	70.6%
**Some bacteria can multiply in the product**							
Chlorine 0.5%	8.6%	13.3%	8.8%		9.8%	13.3%	10.3%
Povidone iodine 10%	21.8%	19.7%	8.9%		18.1%	11.6%	17.1%
ABHR	17.2%	33.2%	24.2%		25.8%	20.7%	25.2%
Household soap	54.8%	67.6%	60.8%		61.8%	58.4%	61.3%
**The product kills all bacteria**							
Chlorine 0.5%	51.0%	46.9%	49.5%		47.2%	60.2%	49.1%
Povidone iodine 10%	20.7%	20.6%	30.7%		23.4%	25.6%	23.7%
ABHR	19.3%	17.3%	17.6%		16.0%	30.5%	18.0%
Household soap	8.6%	6.2%	4.8%		6.2%	9.0%	6.6%
**I do not know**							
Chlorine 0.5%	9.1%	9.5%	15.4%		10.6%	14.5%	11.2%
Povidone iodine 10%	13.5%	25.7%	29.1%		19.2%	43.0%	22.7%
ABHR	8.3%	9.8%	17.6%		8.3%	32.9%	11.7%
Household soap	6.6%	6.2%	11.8%		7.3%	12.4%	8.1%

For Questions 4, 5, 6 and 7, see Supplementary Table 1. Multiple options could be selected for an answer. The proportions (%) represent the ratios of answered options per type of product on the total number of participants for the three study sites and across the professional groups. Abbreviations: ABHR = alcohol-based hand rub, CHU-YO = Centre Hospitalier Universitaire Yalgado Ouédraogo, Ouagadougou, Burkina Faso, CNHU-HKM = Centre National Hospitalier Universitaire Hubert Koutoukou Maga, Cotonou, Benin, UHK = University Hospital of Kinshasa, Kinshasa, DR Congo

Storage of freshly prepared chlorine 0.5% in a transparent squeeze container (presented on a photo), was properly indicated as “*not correct*” by one-third (34.8%) of participants. The proportions of correctly answering participants were largest (but still low) at CHU-YO (42.5%) and lowest at CNHU-HKM (26.2%). The shelf life labeled on the container (1 week) was correctly considered as too long by 37.4% of participants, with again the largest proportion at CNHU-HKM (46.3%). Storage of freshly prepared ethanol 70% presented in well-labeled transparent squeeze container was erroneously replied as “*not correct*” by 39.2% of participants and its shelf life of 1 week was considered as too short by 44.1% of participants.

### Awareness of risks associated with preparation of products and filling of in-use containers

In reply to the case-based question about diluting the chlorine stock solution to a 0.5% working concentration, 56.4% of participants correctly disapproved of the use of tap water, 29.7% of participants agreed to do so and the remaining 13.9% replied they did not know the answer. When presented the above-mentioned mobile handwash station with liquid soap container (Fig. 1), one-third (33.5%) of participants approved the use of tap water for the preparation of in-house liquid soap. The vast majority (82.2%) of participants disapproved the use of non-cleaned utensils for the preparation of chlorine 0.5%.

When questioned about filling in-use containers for hand hygiene in a neonatology ward, over three-quarters (81.9%) replied to do so “*after emptying, washing and disinfecting*”. The remaining 18.3% approved the practice of topping-up of containers.

### Recognizing depicted risk and risk mitigation factors, acceptability of recycled containers

Among scenes which depicted handling of containers (Fig. 1), participants failed to acknowledge the following risks: (i) impregnating compresses while touching the container (40.2% of participants), (ii) touching the rim of the containers when using a stock container to fill an in-use container (37.3%), (iii) storing cotton balls soaked in a container with an antiseptic (44.4%) and (iv) hand-touching the spout of the table-top container’s pump dispenser (34.0%). A total of 29.3% of participants correctly identified all four depicted risks (31.4% and 15.4% for the (para)medical and auxiliary staff, respectively, *p* = 0.003).

When presented with a photo of a recycled soft-drink bottle with perforated screw cap (Figs. 1), 80.6% replied it was unacceptable; 10.7% and 8.9%, respectively answered it was acceptable or “*I do not know*”. Largest proportions of “acceptable” were reported from CNHU-HKM and CHU-YO where respectively 30.4% and 38.1% of auxiliary staff considered the soft-drink bottle acceptable. Two photos depicted risk mitigation of contamination of bar soap. Using small pieces of bar soap and storing in-use bar soap in a perforated receptacle were identified as risk mitigation measures by respectively three-quarters (75.4%) and nearly two-thirds (64.7%) of participants.

### Risk practices: re-use, reprocessing, and refilling of containers

Among a total of 520 participants who declared they were informed about the use of containers in their ward, a minority (10.4%) replied that containers were not reused. Those who reused indicated reuse frequencies of once (11.7% of participants), 2 to 5 times (19.6%), and above (6.3%), the remaining 52.1% of participants declared to reuse them until they were lost or spoiled. The latter practice was less reported from UHK (31.5%) compared to CNHU-HKM and CHU-YO (53.8% and 70.9%, respectively) where it was alike reported from high-risk wards such as in the neonatology and nephrology– dialysis.

Two questions assessed the reprocessing of containers. A first question proposed, for a liquid soap container on a mobile hand-wash station (Fig. 1), to have two containers available: one being used, whereas the other is being reprocessed by washing and drying. Overall, 61.1% of participants replied this was good practice. Proportions were close to two-thirds (66.5% and 65.9%) at CHU-YO and CNHU-HKM, respectively versus half (49.5%) at UHK (*p* = 0.0005). A second question addressed the reprocessing of containers in the participants’ wards. Removing participants replying they did not know or did not reuse containers, two thirds (65.8%) of 444 participants indicated washing and drying and another 20.5% washing without drying, respectively. The remaining (13.7%) declared that washing and drying was not necessary.

### Performance of staff trained in IPC versus staff not trained in IPC

IPC trained participants were more confident about the locally available products compared to non-IPC trained participants (63.7% versus 53.9%, *p* = 0.01). They also scored slightly better for the need to store chlorine 0.5% in a non-transparent container (40.2% versus 30.4%, *p* = 0.01) (Supplementary Table 2). More IPC trained participants considered products as vulnerable to contamination, but differences were small and the rank of vulnerability of products was the same as for the non-trained participants (i.e., considering ABHR more vulnerable than povidone iodine and chlorine). Most striking was the better score for the depicted risk practices: 35.9% of IPC-trained participants correctly recognized all risk practices versus 23.8% of the non-IPC-trained participants, *p* < 0.0001). For the other questions, no significant differences were found, except for the knowledge of Dakin as an antiseptic, which was better among non-IPC trained participants compared to trained ones (78.9% versus 70.1%, *p* = 0.01).

### Post-hoc validation

Answers to questions addressing similar items were congruent. They comprised the risk of using tap water (Q8 and Q14), the stability of freshly prepared chlorine 0.5% (Q11 and Q12) and practices about container reprocessing (Q18 and Q25). Six out of seven questions for which invalid answers were possible had invalid proportions below 5%, the remaining question (Q9, use of non-disinfected items for the preparation of chlorine) had an invalid ratio of 5.7%.

## Discussion

### Summary of findings

The present study surveyed healthcare workers in tertiary care hospitals in sub-Saharan Africa about bacterial contamination of antiseptics, disinfectants, and hand hygiene products. Less than half of the participants had been trained in IPC. Knowledge about products and awareness of risk factors for contamination were limited.

### Training

Less than half of participants had been trained in IPC, and only a minority had been trained in the year before the survey. This is far below the recommendations by the WHO, which prescribe IPC training of the frontline clinical staff at the moment of employment and – for tertiary care centers – annually [35]. Recent studies from university hospitals in Nigeria mentioned low proportions of staff trained in IPC (< 15%) and hand hygiene (24%), respectively [36, 37]. A survey about IPC activities in pediatric hospitals revealed that lack of education was the most coming perceived barrier both in high-income countries and LMIC, with respectively 46% and 25% reporting regular education on IPC [38]. In the present survey, the absence of training among two thirds of medical doctors and trainees at CHU-YO and CNHU-HKM is of huge concern. A previous survey from Uganda has shown that IPC training can be successfully embedded in the medical curriculum and the authors recommend to begin training at the start of medical school [39]. In addition, WHO guidelines recommend, as stated in the Core Component 3 of the Minimum Requirements, an initial training of clinicians and housekeepers upon employment for all healthcare levels, and further annually in the tertiary healthcare centers (WHO2019 – Minimum Requirement).

### Knowledge about products and their vulnerability to contamination, shelf-life, and storage

Although most participants were acquainted with the AS, DI and HH products, they wrongly classified several in-use products as antiseptics or disinfectants respectively. The terms antiseptics and disinfectants are also frequently used interchangeably in medical literature [11, 14]. Besides inappropriate use (e.g., Dakin solution used as a disinfectant) [10], incorrect terminology may hamper product management at the national and hospital level. Eosin 2% water-based solution is used at CHU-YO and CNHU-HKM for skin care [10] and was presently erroneously considered as an antiseptic by nearly half of (para)medical staff. However, it is a chemical dye with no proven antiseptic activity [40]. Of note, eosin 2% solution figures in the national Model List of Essential Medicine of DR Congo and Benin [27, 29].

The healthcare workers’ wrong assumptions that antiseptics and disinfectants kill all bacteria and even are sterile has been noted in high-income countries too [20–22] and is probably a factor fueling inappropriate use [11]. Most striking however was that nearly two-thirds of participants considered ABHR as vulnerable to contamination, a proportion which was larger compared to povidone iodine and chlorine 0.5%. Alcohol-based products are by far the most resistant to contamination (missing only the spore-forming bacteria in their activity spectrum). Conversely, water-based products such as povidone iodine and chlorine 0.5% are more susceptible [11, 14].

Another misconception was the ignorance about storage and shelf life of freshly prepared chlorine 0.5% (overestimated or not known by approximately 60% of participants). Non-stabilized chlorine solutions should be stored in opaque containers as chlorine 0.5% decays by exposure to UV light, and ideally fresh solutions should be prepared daily [32, 41]. By contrast, alcohol-based products are not susceptible to light and have a longer shelf life. The large proportions of incorrect answers are surprising, given the fact that chlorine 0.5% and ABHR have been intensively promoted worldwide as part of containment of the COVID-19 pandemic [32] and hand hygiene [42].

Further, participants were apparently not familiar with the concept of period-after-opening (in-use product stability time). During the aforementioned microbiological survey of AS, DI and HH products, we did not find a day of first opening on any of the products assessed [10]. Defining the period-after-opening has been listed as an outstanding issue in the prevention of bacterial contamination of AS, DI and HH products [11] and is particularly challenging in LMIC, given the harsh conditions in the healthcare environment [10].

### Awareness of risk of bacterial contamination: preparation, recycling, and handling of containers

In LMIC, use of tap water is a well-known source of bacterial contamination of in-hospital prepared products [11, 14], and tap water may be contaminated even if supplied by improved water sources [43, 44]. The relatively large proportion of participants ignoring the risk of tap water (30% who approved it and 10% who replied to do not know it) is in line with previous observations at CHU-YO and CHNU-HKM [10]. By contrast not cleaning the utensils for preparation of chlorine 0.5% was approved by only 6.5% of participants with another 6.4% participants declaring they did not know.

Depicted risks of handling containers were each overlooked by more than one-third of participants. Not recognizing the risk of contamination by physical contact between containers, compresses and hands points to missing insights in the bacterial load in the healthcare environment and the potential transmission routes. Most striking was the high proportion (44.6%) of participants who overlooked the risk of the cotton balls soaked and stored in antiseptics: cork, gauze and cellulose bind and inactivate antiseptics, in particular quaternary ammonium compounds [45].

Access to affordable containers and dispensers in LMIC is challenging [46]. Recycled soft-drink bottles were deemed acceptable by 10.5% of participants and 8.9% declared not to know, but acceptance was above 30% among auxiliary staff at CHU-YO and CNHU-HKM. In the previous microbiological survey at CHU-YO and CNHU-HKM, nearly all (95.5% of 179) of aliquoted non-original in-use containers were recycled and 16.4% of them were recycled soft-drink bottles [10].

### Risk practices: reuse, reprocessing and refilling of containers

Reuse of containers was acknowledged by close to 90% of participants, half of whom reused containers until their end-of-life. The latter practice was most reported from CNHU-HKM and CHU-YO and in line with previous observations [10]. Reuse of patient care items (e.g., oxygen masks, endotracheal and nasal tubes) and personal protective equipment is common in low-resource settings [38]. In a survey about use of ABHR among 39 healthcare facilities worldwide, two-thirds (*n* = 36) reported reusing the dispenser containers, among which one was a high-income country [46].

The high proportion of participants declaring that containers were reprocessed by washing and drying contrast with the 78.8% non-reprocessing of reused containers observed at CHU-YO and CNHU-HKM previously [10]. Likewise, only 18.1% of participants agreed with the proposed option to fill in-use containers in the neonatology ward by topping-up, versus the observation of three-quarters of reused containers filled by topping-up in the previous survey [10]. Knowing that none of the hospitals had a procedure in place for container reprocessing, the apparent discrepancies may have been caused by a survey-related courtesy bias (i.e., replying to the most appropriate answer option). In addition, for participants from CHU-YO and CNHU-HKM, a learning effect from the previous microbiological survey may be probable [10]. Alternatively, it may point to practice justified by underestimation rather than by ignorance of the risk factor. A pre- and post-test survey about best practices for disinfectant use in hospitals in Malaysia showed that practices known as inappropriate may be “institutionalized” and difficult to eradicate [47].

### (Para)medical versus auxiliary staff, differences between IPC trained and non-trained staff

Most findings were consistent across (para)medical and auxiliary staff and differences were relatively small and plausible from their respective experience with the products. Further, compared to (para)medical staff, auxiliary staff had more confidence in the products and less preference for ABHR. Compared to the non-IPC trained participants, IPC-trained participants performed better for selective questions, but differences were small, and knowledge and awareness were still unsatisfactory. Reasons for the overall small differences may be the long period since the training or the fact that the contamination of products was not part of the training program.

### Limitations and strengths

Study limitations mentioned above were courtesy and learning biases. Because of COVID-19 containment measures, surveys were organized in small groups over multiple days, leaving opportunities for information exchange between participants on what questions were asked. Outsourced staff in charge of routine floor cleaning and waste management at CHU-YO and UHK were not included. Finally, the recruitment of participants by voluntary presentation at a meeting point may have attracted the most motivated staff. By absence of updated human resources’ data, staff representativity and response rate could not be calculated. Further, as the survey focused on products used and known to all participants at all three sites, products such as chlorhexidine and quaternary ammonium compounds (known for their vulnerability to contamination [11]) were not addressed.

Among the strengths were the context and language adapted questionnaire, based on extensive literature review and on-site study and observations [10, 14]. The questionnaire was piloted in a LMIC audience and its large sample size allowed to address relevant subgroups. The digitalized application was easy to handle and prevented retrograde correction of answers. The study team was available on-site for assistance. Internal validation parameters were satisfactory. To our knowledge, this is the first Knowledge, Awareness and Practice survey addressing specifically bacterial contamination of AS, DI and HH products. Previous surveys about Infection Prevention & Control in sub-Saharan Africa mostly addressed IPC in general and hand hygiene in particular [36, 39, 48].

### Relevance, outstanding issues and future research, generalizability

They present findings highlighted gaps in knowledge, limited awareness, and inappropriate practices, conducive to bacterial contamination of AS, DI and HH products. They refine and extend previous findings obtained by interview and observations [10] and can guide training and implementation. They further confirm that risk factors for contamination of products exist across the hospital and affect also high-risk wards.

Training is one of the WHO Core Components of implantation of IPC in healthcare facilities [49]. In line with WHO recommendations [1, 35, 50], the present results point to the need to address, besides frontline clinical staff and cleaners, also pharmacy, procurement, and logistics staff in the educational activities about AS, DI and HH products. Further, to improve adherence, training should also address understanding of the healthcare facility environment’s microbiology and transmission routes. As none of the three hospitals had procedures about AS, DI and HH products in place, generic procedures and guidelines (as done for environmental cleaning [51]) are welcome. Given the persistent nature of some risk practices (soaking cotton balls, using tap water, topping-up [11]), there is a definite need for refresher trainings, workplace reminders, administrative monitoring, and an institutional safety climate in line with the multimodal strategy of implementation of IPC measures [49].

The present findings also confirm outstanding issues [11] such as defining the period-after-opening of products and the need for field-adapted containers. The table-top pump dispenser’s container preferred by the majority of participants is notably difficult to clean, hampering disinfection [46, 52, 53]. Future research should include behavior studies to understand and tackle the existence of inappropriate practices despite notions or knowledge of risk. Further, market mechanisms should be explored to assure affordable access to containers or containers which can withstand autoclave-based sterilization. Finally, the participants’ explicit preference for liquid soap over ABHR and their erroneous perception of the vulnerability to contamination of the latter product may constitute useful information for the WHO hand hygiene campaigns.

The survey’s sites were tertiary care hospitals in sub-Saharan Africa with functional microbiology laboratories, pharmacies, and IPC services. Knowledge, awareness, and practices may be worse in the other healthcare facilities, particularly those which are rural and remote.

## Conclusion

The present study in three tertiary care hospitals in sub-Saharan Africa showed that, despite end-users’ acquaintance and confidence with locally used AS, DI and HH products, knowledge about the products was inadequate, with misconceptions about the vulnerability to bacterial contamination. Likewise, there was limited awareness about risks of contamination at preparation and in-use handling. Reported practices conducive to contamination were indefinite reuse of recycled containers, and to a lesser extent reprocessing and topping-up. Findings were consistent across the three sites, professional groups ((para)medical and auxiliary staff) and wards. Coverage of training in IPC was far below the WHO recommendation and risk practices occurred also in areas with vulnerable patients (neonatology, dialysis). The findings can sensitize stakeholders’ awareness and interest and guide tailored training and implementation of risk mitigation measures in healthcare facility and national IPC action plans.

### Electronic supplementary material

Below is the link to the electronic supplementary material.

Supplementary Material 1

Supplementary Material 2

## Data Availability

Data supporting the present work results can be found in Supplementary Document 1 submitted with this work.

## Abbreviations

AS: Antiseptics
CHU-YO: Centre Hospitalier Universitaire Yalgado Ouédraogo
CNHU-HKM: Centre National Hospitalier Universitaire Hubert Koutoukou Maga
DI: Disinfectants
DR Congo: Democratic Republic of the Congo
HH: Hand hygiene
IPC: Infection prevention and control
KAP: Knowledge, awareness, and practice
LMIC: Low- and middle-income countries
UHK: University Hospital of Kinshasa
