# Supplementary material for: Knowledge, awareness, and risk practices related to bacterial contamination of antiseptics, disinfectants, and hand hygiene products among healthcare workers in sub-saharan Africa: a cross-sectional survey in three tertiary care hospitals (Benin, Burkina Faso, and DR Congo)
Source: Antimicrob Resist Infect Control. 2024 Apr 16;13:44. doi: 10.1186/s13756-024-01396-3 (PMC11020199; doi:10.1186/s13756-024-01396-3)
Supplement: Supplementary file 2 — Supplementary Material 2 [file 13756_2024_1396_MOESM2_ESM.docx]

**Supplementary Table 1.** **Overview of the questionnaire used to assess healthcare workers' knowledge, awareness, and practices about bacterial contamination of antiseptics, disinfectants, and hand hygiene products.** For questions marked with a "*", multiple options for answer were possible. Questions Q1 and Q17 were removed from data analysis (see text). Abbreviations:

| **Question category and Nr.** | **Question (translated from French)** | **Answer options for** | | | | | | | | | References |
| --- | --- | --- | --- | --- | --- | --- | --- | --- | --- | --- | --- |
| I | How old are you? | □ < 18 years | □ 31 to 40 years old | | | | | | □ 51 to 60 years | |  |
|  |  | □ 18 to 30 years | □ 41 to 50 years | | | | | | □ > 60 years | |  |
| II | What is your gender? | □ Male | | | □ Female | | | | | |  |
| III | How many years of hospital experience do you have? | □ < 1 year  □ 1 to 5 years | | □ 6 to 10 years  □ >10 years | | | | | | □ not applicable |  |
| IV | Professional profile | Different options per country | | | | | | | | |  |
| V | Hospital or center | □ CHU-YO □ CNHU-HKM □ UHK | | | | | | | | |  |
| VI | Hospital Ward | Different options per country | | | | | | | | |  |
| VII | When was your last training course in infection prevention and control (”*hygiene hospitalière*”)? | □ ≤ 1 year | | | □ I do not remember | | | | | |  |
|  |  | □ > 1 year | | | □ Never had any training | | | | | |  |
| VIII | When was your last training course specifically on antiseptics and disinfectants? | □ ≤ 1 year | | | □ I do not remember | | | | | |  |
|  |  | □ > 1 year | | | □ Never had any training | | | | | |  |
| IX* | What experience do you have with antiseptics and disinfectants? You can indicate several options. | □ Hand hygiene | | | □ Preparation of antiseptic/disinfectant products | | | | | |  |
|  |  | □ Wound dressing | | | □ Supply of antiseptic/disinfectant products | | | | | |  |
|  |  | □ Cleaning of care surfaces | | | □ I have never used them | | | | | |  |
|  |  | □ Antisepsis of the patient's skin | | | □ Other (specify): ... | | | | | |  |
|  |  | □ Cleaning medical tools (e.g., stethoscopes, thermometers) | | |  | | | | | |  |
| Q1 | What concentration of alcohol is indicated for hand disinfection and hygiene? | □ 95% | | | □ None of the above concentrations is appropriate | | | | | |  |
|  |  | □ 70% | | | □ Other concentration (specify on next page) | | | | | |  |
|  |  | □ 50% | | | □ I do not know | | | | | |  |
| Q2* | Tick all the products used for skin disinfection in the list below. | □ 70% ethanol □ 0.5% bleach □ Dakin □ Methanol | | | | □ Betadine □ Glycerin □ 70% Isopropanol □ Eosin | | | | | [7,11,29] |
| Q3* | Tick all the products in the list below that are suitable for disinfecting equipment and inert (non-living) surfaces. | □ 70% ethanol □ 0.5% bleach □ Dakin □ Methanol | | | | □ Betadine □ Glycerin □ 70% Isopropanol □ Eosin | | | | | [11,14,29] |
| Q4* | Which statement do you think is correct? You can choose several answers | □ 10% povidone-iodine solution (Betadine) kills all bacteria (microbes). | | | | | | | | | [7,13,49,50] |
|  |  | □ Some bacteria (microbes) are resistant to 10% povidone-iodine (Betadine) and can survive in it. | | | | | | | | |  |
|  |  | □ Some bacteria (microbes) can multiply in 10% povidone-iodine (Betadine). | | | | | | | | |  |
|  |  | □ I do not know. | | | | | | | | |  |
| Q5* |  | □ Some bacteria (microbes) are resistant to alcohol-based hand rub and can survive in it. | | | | | | | | |  |
|  |  | □ The alcohol-based hand rub kills all bacteria (microbes). | | | | | | | | |  |
|  |  | □ Some bacteria (microbes) can multiply in alcohol-based hand rub. | | | | | | | | |  |
|  |  | □ I do not know. | | | | | | | | |  |
| Q6* |  | □ Bleach 0.5% kills all bacteria (microbes). | | | | | | | | |  |
|  |  | □ Some bacteria (microbes) are resistant to 0.5% bleach and can survive in it. | | | | | | | | |  |
|  |  | □ Some bacteria (microbes) can multiply in bleach. | | | | | | | | |  |
|  |  | □ I do not know. | | | | | | | | |  |
| Q7* |  | □ Certain bacteria (microbes) are capable of multiplying in household soap. | | | | | | | | | [7,14,51] |
|  |  | □ Some bacteria (microbes) are resistant to household soap and can survive in it. | | | | | | | | |  |
|  |  | □ Household soap kills all bacteria (microbes). | | | | | | | | |  |
|  |  | □ I do not know. | | | | | | | | |  |
| Q8 | A pharmacist uses tap water to dilute alcohol and bleach. His colleague asks, "Doesn't this water contain bacteria (microbes)?" The pharmacist replies: "Even if there are bacteria in the water, when mixed with the alcohol or bleach, they are killed immediately". Is the pharmacist right? | □ Yes □ No □ I do not know | | | | | | | | | [7,29,45] |
| Q9 | Yesterday, the pharmacist diluted bleach using a set of materials (measuring cup, containers, etc.). He did not clean the materials after use. Today, he is going to dilute the bleach with the same materials. Did he proceed correctly? | □ Yes, you only need to wash the materials if you use the same materials for different products.  □ No, the materials must be washed each time they are used.  □ I do not know | | | | | | | | | [7] |
| Q10 | It is July 2020 (look at the picture). The expiration date on your container of Betadine is December 2020. You opened it in January 2020, are you still going to use it?  (Photo-based question) | □ Yes □ No □ I do not know | | | | | | | | | [7,36] |
| Q11 | To disinfect treatment rooms, healthcare staff use a 0.5% dilution of bleach. How long do you use this dilution? | □ 1 day □ 2 days □ 1 week | | | | □ More than a week □ None of the answers is applicable □ I do not know | | | | | [1,29,52] |
| Q12a* | In a hospital ward, there are two containers of bleach diluted to 0.5% (see photo).  On the label, the nurse wrote: Production date: 21/06/2020, Expiry date: 27/06/2020 Which statement do you agree with? You may indicate 1 or 2 answers, or none. (Photo-based question) | □ Bleach is not stored correctly.  □ The bleach expiration date is too long.  □ Neither of these statements  □ Other (specify on the next page the appropriate duration for use of the diluted bleach solution) | | | | | | | | | [12,29,52] |
| Q12b* |  |  |  |  |  |  |  |  |  |  |  |
| Q13a* | In a hospital ward, there are two containers of 70% ethanol (see photo). On the label the nurse wrote: Production date: 21/06/2020 Expiry date: 27/06/2020 Which statement do you agree with? You may indicate 1 or 2 answers, or none. (Photo-based question) | □ Ethanol is not stored correctly.  □ The expiration date of the ethanol solution is too short. □ Neither of these statements  □ Other (specify on the next page the appropriate duration for use of the ethanol solution) | | | | | | | | | [7,36] |
| Q13b* |  |  |  |  |  |  |  |  |  |  |  |
| Q14a* | The hospital has a mobile hand-washing station (see picture). Antibacterial soap is prepared on site and distributed into bottles. Which of the options below are good practices? You can choose several answers. (Photo-based question) | □ Soap is prepared with tap water □ The soap container is used for a maximum of 1 week.  □ There are 2 containers of soap: while one is in use, the other is emptied, washed, rinsed, and dried. | | | | | | | | | [7,11,14,47] |
| Q14b* |  |  |  |  |  |  |  |  |  |  |  |
| Q14c* |  |  |  |  |  |  |  |  |  |  |  |
| Q15* | On the images of the containers below used for alcohol-based hand rub, tick the box corresponding to the type of container you prefer. You can choose several answers  (Photo-based question) | □ Squeeze container □ Container with pump dispenser □ Container with flip top snap cap dropper □ Container with screw cap | | | | | | | | | [7] |
| Q16 | If you reuse old containers for liquid soap, alcohol, or bleach, how often do you reuse them in your ward? | □ 1 time  □ 2 to 5 times  □ 6 to 10 times  □ > 10 times | | | | □ Until it is lost or spoiled  □ We do not reuse containers  □ I do not know | | | | | [7,41,47,53] |
| Q17 | In the neonatal ward, the number of times a bottle of antiseptic can be reused is limited. How many times do you consider acceptable? | □ 1 time | | | | | □ >10 times | | | |  |
|  |  | □ 2 to 5 times | | | | | □ Until it is lost or spoiled | | | |  |
|  |  | □ 6 to 10 times | | | | | □ I do not know | | | |  |
| Q18 | If you reuse old containers, how do you process them before reusing them in your ward? | □ We wash and dry the containers □ We wash the containers but do not dry them □ We do not wash containers □ We do not reprocess containers □ I do not know | | | | | | | | | [7,9,41,47,53] |
| Q19 | A container (see photos) has been reused to fill with liquid soap for hand hygiene. Look at the photos and say if this way of recycling a container is acceptable. (Photo-based question) | □ Yes □ No □ I do not know | | | | | | | | |  |
| Q20 | Are you confident about the quality of the antiseptics and disinfectants used in your ward? | □ I have absolute confidence □ I have confidence □ I have sufficient confidence | | | | | | □ I have a little confidence  □ I have no confidence | | |  |
| Q21a* | The figures show practices concerning antiseptics in hospital wards. Indicate the practice that is NOT correct. You may indicate more than one figure.  (Photo-based question) | □ Impregnating a cotton pad with antiseptic by touching it directly to the container content and rim | | | | | | | | | [29] |
| Q21b* |  | □ Filling a small container with a large stock container while touching the rims of both bottles. | | | | | | | | |  |
| Q21c* |  | □ Storage of cotton pads soaked in a container with antiseptic. | | | | | | | | | [9,29] |
| Q21d* |  | □ Touching the spout of the dispenser’s pump while dispensing products for hand hygiene | | | | | | | | | [29] |
| Q22 | In case you only have bar soap available, which of the following practices is recommended for infection control (see also photos)? (Photo-based question) | □ Use the large soap bar as it is.  □ Cut the large bar of soap into smaller pieces and use one piece after the other.  □ I do not know | | | | | | | | | [7] |
| Q23 |  | □ Place the bar soap in an open, perforated soap dish.  □ Place the bar soap in an unperforated bowl.  □ I do not know | | | | | | | | |  |
| Q24 | What type of hand hygiene product do you prefer in your department? | □ Bar soap □ Plain liquid soap □ Plain powder soap | | | | □ Antiseptic liquid soap □ Alcohol-based hand rub solution □ No preference | | | | | [7,51] |
| Q25* | Which product do you use for hand hygiene in your ward? You may indicate more than one answer. | □ Antiseptic liquid soap □ Non-antiseptic liquid soap (plain) □ Alcohol-based hand rub solution | | | | | | □ Any of the three answers above □ Other (specify on next page) □ I do not know | | | [7] |
| Q26 | In a hospital ward (*e.g*., neonatology), the small containers of liquid soap are almost empty. You are going to refill the small containers with soap from the large (5-liter) stock container. How do you proceed? | □ First you empty, wash, and disinfect the small containers, then you add the soap from the stock.  □ You do not empty or wash the small containers before refilling them.  □ I do not know | | | | | | | | |  |

**Supplementary Table 2. Overview of participants' (n = 617) answers to selected questions about knowledge, awareness and practices related to antiseptics, disinfectants, and hand hygiene products, matched for being trained in IPC (n = 281) or not (n = 336).** Abbreviations: ABHR = alcohol-based hand rub, AS = antiseptics, DI = disinfectants, HH = hand hygiene, NS: not significant.

|  | **All participants (%)** | **IPC trained (%)** | **IPC not trained (%)** | **All participants (n)** | **IPC trained (n)** | **IPC not trained (n)** | **p-value** |
| --- | --- | --- | --- | --- | --- | --- | --- |
| Familiarity with AS DI and HH products | | | | | | | |
| Participants having confidence in the products used in the ward | 58.3% | 63.7% | 53.9% | 360 | 179 | 181 | 0.01 |
| Participants preferring alcohol-based hand rub for hand hygiene | 33.1% | 31.3% | 31.5% | 204 | 88 | 106 | NS |
| Participants preferring liquid soap for hand hygiene | 64.3% | 65.8% | 63.1% | 397 | 185 | 212 | NS |
| Participants preferring the table-top pump-dispenser container | 87.8% | 87.5% | 88.1% | 542 | 246 | 296 | NS |
| Knowledge about bacterial contamination | | | | | | | |
| Participants indicating Dakin solution as antiseptic | 74.9% | 70.1% | 78.9% | 462 | 197 | 265 | 0.01 |
| Participants indicating ethanol 70% as antiseptic | 43.9% | 47.0% | 41.4% | 271 | 132 | 139 | NS |
| Participants indicating eosin as antiseptic | 43.3% | 43.4% | 43.2% | 267 | 122 | 145 | NS |
| Participants indicating Dakin solution as disinfectant | 22.7% | 21.0% | 24.1% | 140 | 59 | 81 | NS |
| Participants replying that bacteria are resistant to and can survive in household soap ^a^ | 70.6% | 77.2% | 63.1% | 429 | 217 | 212 | 0.0002 |
| Participants replying that bacteria are resistant to and can survive in ABHR | 65.0% | 65.5% | 58.9% | 382 | 184 | 198 | NS |
| Participants replying that bacteria are resistant to and can survive in chlorine 0.5% | 37.4% | 36.7% | 35.1% | 221 | 103 | 118 | NS |
| Participants replying that bacteria are resistant to and can survive in povidone iodine | 51.0% | 55.2% | 43.5% | 301 | 155 | 146 | 0.005 |
| Participants indicating the 24h shelf life of freshly prepared chlorine 0.5% | 42.6% | 48.4% | 37.8% | 263 | 136 | 127 | 0.009 |
| Participants agreeing with a 1-week shelf-life of in-use locally prepared liquid soap | 30.3% | 31.0% | 29.8% | 187 | 87 | 100 | NS |
| Participants agreeing to use povidone-iodine in-use in the last seven months | 40.7% | 37.4% | 43.5% | 251 | 105 | 146 | NS |
| Participants awareness of the need to store chlorine 0.5% in a non-transparent container | 34.8% | 40.2% | 30.4% | 215 | 113 | 102 | 0.01 |
| Awareness of the risk of bacterial contamination | | | | | | | |
| Participants approving the use of tap water for preparation of chlorine 0.5% | 29.7% | 30.2% | 29.2% | 183 | 85 | 98 | NS |
| Participants not recognizing the (depicted) risk of storing cotton balls soaked in AS | 44.4% | 42.3% | 46.1% | 274 | 119 | 155 | NS |
| Participants recognizing all 4 depicted risk handling of antiseptics | 29.3% | 35.9% | 23.8% | 181 | 101 | 80 | < 0.0001 |
| Participants accepting the use of a recycled soft drink bottle as a storage container | 10.7% | 9.6% | 11.6% | 66 | 27 | 39 | NS |
| Participants agreeing to reprocess liquid soap containers by washing and drying | 61.1% | 63.0% | 59.5% | 377 | 177 | 200 | NS |
| Participants declaring to fill an in-use container by topping-up (neonatal ward) ^b^ | 18.3% | 17.4% | 14.9% | 99 | 49 | 50 | NS |
| Risk practices conducive to bacterial contamination | | | | | | | |
| Participants declaring, they reused containers indefinitely ^c^ | 52.1% | 44.1% | 43.8% | 271 | 124 | 147 | NS |
| Participants declaring, they reprocessed containers by emptying, washing, and disinfecting ^d^ | 81.9% | 71.5% | 72.3% | 444 | 201 | 243 | NS |

^a^ Percentage calculated after removing invalid answer combination (n = 9).

^b^ Percentage calculated after removing answering “*I do not know*” (n = 7).

^c^ Percentage calculated after removing participants who did not know about the reprocessing of containers in their wards (n = 97).

^d^ Percentage calculated after removing participants answering “*I do not know*” (n = 75).
